# An Evaluation of the Role of Oxidative Stress in Non-Obstructive Coronary Artery Disease

**DOI:** 10.3390/jcdd9020051

**Published:** 2022-02-04

**Authors:** Nurnajwa Pahimi, Aida Hanum Ghulam Rasool, Zulkefli Sanip, Nur Adilah Bokti, Zurkurnai Yusof, W. Yus Haniff W. Isa

**Affiliations:** 1 Pharmacology Vascular Laboratory, Department of Pharmacology, School of Medical Sciences, Universiti Sains Malaysia, Kota Bharu 16150, Malaysia; najwapahimi@student.usm.my (N.P.); aida.rasool@yahoo.com (A.H.G.R.); 2 Cardiology Unit, Department of Medicine, School of Medical Sciences, Universiti Sains Malaysia, Kubang Kerian 16150, Malaysia; nuradilahbokti@usm.my (N.A.B.); zurkurnai@gmail.com (Z.Y.); 3 Department of Internal Medicine, Hospital Universiti Sains Malaysia, Kubang Kerian 16150, Malaysia; 4 Central Research Laboratory, School of Medical Sciences, Universiti Sains Malaysia, Kubang Kerian 16150, Malaysia; zulkefli@usm.my

**Keywords:** coronary artery disease, non-obstructive coronary artery disease, oxidative stress

## Abstract

Approximately half of all women presenting to the emergency department with angina chest pain do not have obstructive coronary artery disease (CAD) on coronary angiography. This condition is termed non-obstructive coronary artery disease (NOCAD), and includes ischemia with no obstructive coronary artery disease (INOCA) and myocardial infarction with non-obstructive coronary arteries (MINOCA). Oxidative stress has been reported to be involved in the development and progression of CAD. However, a scarcity of studies has assessed a correlation between oxidative stress and NOCAD. Thus, a literature review was performed of available reports on the role of oxidative stress in NOCAD. Possible mechanisms involved in oxidative stress that may contribute to NOCAD were identified and evaluated. A key finding of this literature review was that oxidative stress caused vasoconstriction and endothelial damage, and this results in coronary microvascular dysfunction and vasospasm, which, in turn, lead to the pathogenesis of NOCAD.

## 1. Introduction

Cardiovascular diseases cause approximately one third of all deaths worldwide [1]. According to the American Heart Association, coronary artery disease (CAD) ranks as the most prevalent of all types of cardiovascular disease [1]. CAD remains the primary cause of death in Malaysia and accounted for approximately 15% of all mortality in 2019, and this figure is increasing steadily [2]. An increasing number of individuals diagnosed with CAD live with chronic disability and poor quality of life [3,4]. In addition, CAD is associated with significant social and economic costs globally [5]. Accordingly, it represents a major challenge to healthcare systems and societies worldwide.

The most common symptom of CAD is angina pectoris [6], which affects approximately 112 million people globally [7]. Angina pectoris is caused by myocardial ischemia, which creates a temporary and reversible imbalance between myocardial blood supply and oxygen demand [7]. This may occur in the presence of a fixed stenosis or as a result of vasospasm of the coronary arteries [8]. A subset of patients who present with symptoms of angina pectoris do not present with obstructive lesions on coronary angiogram, and this condition is referred to as non-obstructive coronary artery disease (NOCAD). Currently, the proposed mechanisms or factors that may explain the pathogenesis of NOCAD include oxidative stress [9,10,11,12,13], coronary vasospasm [14,15,16], and microvascular endothelial dysfunction [11,17,18,19,20,21,22]. In the current study, a review of the literature on the topic was performed to evaluate the possible role of oxidative stress in the development and progression of NOCAD, with a view to establishing optimal management approaches to the disease, minimizing the disease burden, and improving the quality of life of people with NOCAD.

## 2. Oxidative Stress

Oxidative stress, defined as a disturbance to the balance between the production of reactive oxygen species (ROS) and antioxidant defense, which leads to tissue injury, appears to be involved in the pathogenesis of many diseases, including cardiovascular disease [23,24,25]. ROS include free radicals, such as superoxide anion and hydroxyl radicals, as well as nonradical molecules, such as hydrogen peroxide and singlet oxygen [23]. Free radicals are formed in large amounts as byproducts of many biochemical processes. ROS are a natural byproduct of normal oxygen metabolism and play an important role in cell signaling and cellular homoeostasis under normal circumstances [26]. 

The overexpression of ROS (i.e., in excess of antioxidant defense) can damage cell structures, including carbohydrates, nucleic acids, lipids, and proteins, which alters their functions [26]. Antioxidants are present in cells to prevent the damage incurred by oxidative stress. The scavenging of excess ROS is achieved by the antioxidative defense system, which comprises enzymatic antioxidants (i.e., superoxide dismutase (SODs), catalase, and glutathione peroxidase) [12] and non-enzymatic antioxidants (i.e., glutathione (GSH), polyphenols, and vitamins) [27]. ROS are key players in the induction of oxidative stress. They oxidize lipids, proteins, and DNA by repeated free radical attacks, thus causing tissue damage, which leads to lipid peroxidation, protein denaturation, and DNA mutation [25,28].

### Sources of Reactive Oxygen Species

ROS are produced from either endogenous or exogenous sources. Endogenous sources of ROS, for example, mitochondria, peroxisomes, and endoplasmic reticulum, all have high oxygen consumption levels [29]. Exogenous sources of ROS include environmental pollution, radiation, cigarette smoking, certain foods, and drugs [30]. Major examples of ROS include superoxide, hydrogen peroxide, hydroxyl anions, hydroxyl radicals, and hypochlorous acid [31]. 

In addition, cellular sources of ROS are divided into two main groups—those that result from biological processes, mainly produced by mitochondrial oxidative metabolism where ROS are released as a byproduct or waste product of various reactions, and those that are released as a result of cellular responses to cytokines, xenobiotics, or bacterial invasion. Thus, ROS are produced through molecular synthesis or breakdown, or as part of a signal transduction pathway or cellular defense mechanism [32]. 

Most cellular ROS sources are partially reduced forms of oxygen and its derivatives, which result from a one-electron oxygen reduction reaction that produces a superoxide anion. Superoxide, which is also generated by cellular redox enzymes, such as nicotinamide adenine dinucleotide phosphate oxidase, stimulated via receptor-mediated mechanisms, is a byproduct of mitochondrial respiration [33]. Hydrogen peroxide, generated from the dismutation of superoxide, is produced due to various enzymatic reactions, and this occurs spontaneously or is catalyzed by SOD [34]. Hydrogen peroxide is also produced by a two-electron enzymatic reduction of molecular oxygen by various oxidases (e.g., xanthine oxidase) [35]. Superoxide reacts rapidly with nitric oxide (NO) to form peroxynitrite [36].

ROS are associated with physiological and pathological biochemical reactions. Under physiological conditions, ROS act as signaling molecules and play a role in maintaining the redox balance. ROS are an important regulator of redox signaling in the heart as they are involved in multiple physiological processes, including differentiation, proliferation, and cardiac excitation–contraction coupling [37]. ROS act by activating ryanodine receptor Ca^2+^ release channels in the junctional sarcoplasmic reticulum in the cardiac muscle, which increases the microscopic release of the Ca^2+^ and modulates the excitation–contraction coupling process. In the heart, the redox balance is critical to maintaining the proper functioning of cellular vital functions, such as excitation–contraction coupling, cell differentiation, homeostasis, and functions related to the stress response pathway, such as adaptation to ischemia [32,38,39,40].

Under pathological conditions, increased ROS levels induce excessive oxidative stress, critically contributing to the pathogenesis and development of various chronic and degenerative diseases, such as cardiovascular disease, diabetes mellitus, Alzheimer’s disease, chronic kidney disease, multiple sclerosis, inflammation, aging, and cancer [25,41,42,43,44]. Oxidative stress is associated with cardiovascular risk factors and adverse outcomes [45,46] related to the vascular system. Increased levels of oxidative stress enhance the formation of oxidized low-density lipoprotein (LDL) which is rapidly taken up by macrophages in the arterial wall, thus transforming these cells into foam cells [47,48,49]. Macrophage-derived foam cells are believed to play an important role in the development and progression of atherosclerosis through the production of various bioactive molecules [50,51]. Increased levels of ROS also degrade vascular NO, which leads to a reduction in NO bioavailability, and, in turn, vasoconstriction [52]. 

Kugiyama et al. reported high levels of plasma-oxidized LDL in patients with coronary artery spasm, compared to the control group. High levels of oxidized LDL correlate with the constrictor reaction of the coronary arteries in response to intracoronary infusion of acetylcholine [53]. Oxidized LDL stimulates the production of endothelin and may contribute to the development of coronary-artery spasm [54]. Thus, higher levels of oxidized LDL have been identified as a risk factors for coronary spasm, as well as having a potential role in its pathogenesis [53].

## 3. Consequences of Oxidative Stress

The endothelium is a single layer of simple squamous cells that lines the interior sur-face of the blood and lymphatic vessels, positioned between the circulating blood and the vascular wall; it was once considered to function only as a simple passive barrier between the circulating blood and surrounding tissues [55]. However, emerging evidence has shown that the endothelium has multiple functions that are crucial to vascular physiology. It plays an important role in the regulation of vascular tone, platelet activity, leukocyte adhesion, and thrombosis [55]. In addition, it acts as a major regulator of vascular homeostasis, which is responsible for several vasoprotective actions, such as vasodilation, the reduction in smooth muscle cell growth, and inhibition of the inflammatory response [56]. It also controls vascular permeability and prevents platelet aggregation. The endothelium provides endothelium-dependent vasorelaxation in response to vascular stress [57]. It also regulates vascular tone by releasing mediators, such as NO, prostacyclin, and endothelium-derived hyperpolarizing factor, or by causing vasoconstriction through the release of thromboxane A2, endothelin-1, or free radicals [58].

A healthy endothelium reacts to mechanical stimuli, chemical factors, and humoral agents by producing several mediators, such as NO, to maintain vasomotor tone and the structural integrity of vessels. NO is the predominant mediator of vascular tone in conduit vessels and is degraded by free radicals present in conditions of increased oxidative stress [57]. ROS has been shown to cause NO degradation, thereby causing vasoconstriction and endothelial damage [14]. Major sources of NO are generated by endothelial NO synthase, a cytochrome p450 reductase-like enzyme that uses tetrahydrobiopterin to form NO from L-arginine [57]. NO diffuses into the underlying vascular smooth muscle, which induces smooth muscle relaxation and vasodilation in response to endothelium-dependent vasodilators, such as acetylcholine [59].

### Coronary Artery Spasm

Oxidative stress, endothelial dysfunction, and low-grade chronic inflammation con-tribute to the pathogenesis of coronary artery spasm. Yamada et al. suggested that oxidative stress by thiol oxidation caused coronary artery spasm, resulting in impaired endothelium-dependent vasodilation. The study measured vascular responses in the isolated coronary arteries of two types of rodents (SMP30 KO and wild-type mice) to acetylcholine and sodium nitroprusside [60]. 

Thiol oxidation is involved in various biological processes; for example, it stimulates changes in protein conformation by transforming free thiols into sulfenic acids, sulfinic acids, sulfonic acids, and disulfide bridges [60]. It has been suggested that thiol oxidation by oxidative stress may trigger coronary artery spasm due to the impairment of endothelium-dependent vasodilation and coronary artery smooth muscle hypercontractility, which leads to increased coronary smooth muscle Ca^2+^ sensitivity through Rho-kinase activation, ultimately resulting in hypercontraction [61]. In addition, endothelial dysfunction may contribute to coronary artery spasm, which may be mediated by impaired endothelial NO synthase activity or smooth muscle cell contraction with Rho-kinase activation at the spasm site [60].

Thioredoxin is a class of small redox proteins that contains redox-active dithiol/disulfide at the active site. It has a variety of biological functions, including cytoprotection against oxidative stress. Increased thioredoxin production occurs to lessen cellular injury caused by ROS in patients with myocardial ischemia. Thioredoxin is a scavenger of ROS [62]. High levels of thioredoxin could indicate active myocardial ischemia activity [63]. Miyamoto et al. demonstrated that thioredoxin plasma levels were higher in patients with coronary spastic angina, compared to those in the chest pain syndrome group. A large proportion of the patients (i.e., 170/240) who were diagnosed with <25% stenosis on angiography underwent cardiac catheterization. The participants were further subdivided into two groups. The first group comprised participants with coronary spastic angina and normal coronary angiography (*n* = 84 patients); the second group consisted of participants with chest pain syndrome (*n* = 86 patients) (i.e., the absence of coronary spasm provoked in the coronary arteries). Increased thioredoxin levels correlated with high disease activity, indicated by the frequency of chest pain attacks [64]. 

Nishihira et al. suggested that thioredoxin was upregulated in patient with unstable angina pectoris due to increased oxidative stress and that it was associated with intraplaque hemorrhage and thrombus formation [65]. Thioredoxin production is induced by oxidative stress, and it is secreted by endothelial cells [66]. In most situations, the cells will activate an adaptive mechanism to protect them from the harmful effects of oxidative stress; however, oxidative stress increases if the oxidant level is persistently greater than the adaptive capability of the cells [67]. Thioredoxin, a sensitive indicator of oxidative stress, could be a potential marker for antioxidative therapy.

Tanabe et al. reported that the levels of serum nitrotyrosine, as oxidative marker, significantly increased after coronary artery vasospasm induced by the acetylcholine provocation test, performed with an injection of acetylcholine through the catheter into the right coronary artery (a dose of either 20 µg or 50 µg) and into the left coronary artery (dose of 50 µg or 100 µg), both administered within a minute. Coronary angiography was performed three minutes after each injection. A comparison was performed of the serum levels of 4-hydroxynonenal (HNE) and nitrotyrosine in 30 patients with a suspected diagnosis of vasospastic angina following the administration of the acetylcholine provocation test. The HNE levels did not change after the test; yet there was increase in nitrotyrosine concentration in the group of patients who were positive for vasospastic angina [59]. This suggests that peroxynitrite contributed to the pathogenesis of coronary artery vasospasm as nitrotyrosine is recommended to assess oxidative stress in coronary artery spasm rather than HNE. Nitrotyrosine is produced by tyrosine nitration, which is mediated by reactive nitrogen species, including peroxynitrite anion and nitrogen dioxide [68]. It is formed in the presence of the active metabolite, NO. In addition, nitrotyrosine is considered to be a marker for cell damage, inflammation, and NO generation [59].

In their study, Ohba et al. reported that coronary artery spasm was identified by distinctive clinical features in patients with non-obstructive coronary arteries and coronary endothelial dysfunction assessed using the acetylcholine provocation test. The study included 370 stable angina patients with suspected non-obstructive coronary arteries (<50% stenosis) on coronary angiography who underwent the acetylcholine provocation test, along with the simultaneous measurement of transcardiac lactate production and quantitative coronary blood flow [15]. Increased lactate levels in the coronary circulation indicates signs of myocardial ischemia [69,70].

In the Ohba et al. study, 50 of the 370 patients with microvascular coronary artery spasm demonstrated significant impairment of the endothelium-dependent vascular response using a quantitative assessment of coronary blood flow (i.e., decreased blood flow on the acetylcholine provocation test) [15]. The acetylcholine provocation test is an endothelium-dependent coronary reactivity test used to assess endothelial function and coronary artery spasm. Acetylcholine causes vasodilation when the endothelium is intact by releasing NO; by contrast, acetylcholine produces vasoconstriction when endothelial function is impaired [71,72]. Ludmer et al. determined that paradoxical vasoconstriction induced by acetylcholine took place during the development of coronary atherosclerosis. The authors suggested that an abnormal vascular response to acetylcholine reflected the impairment of endothelial function [72,73], hence contributing to pathogenesis of the coronary artery spasm.

## 4. Roles of Oxidative Stress in Non-Obstructive Coronary Artery Disease

Over the past three decades, evidence has accumulated to elucidate the contribution of oxidative stress to the development of CAD (Table 1); however, few reports are available on role of oxidative stress in patients with NOCAD (Table 2). Şahin et al. reported that oxidative status and C-reactive protein levels in patients with NOCAD were higher than those in the control group. The study compared three groups of subjects (*n* = 80). The first group comprised 33 patients with typical angina who had normal coronary arteries on angiography and a positive stress test; the second group comprised 27 patients with atypical angina who had no evidence of vasospastic angina, normal coronary arteries on angiography, and a negative stress test, and the last group was the control (i.e., healthy subjects). The authors reported that antioxidant status was lower in patients with NOCAD, compared to the control group [12]. In addition, an increase in oxidative stress was linked to endothelial microvascular dysfunction [74]. In the study, oxidative status was measured by total oxidant status, lipid hydroperoxide levels, and an oxidative stress index; antioxidative status was measured by total antioxidant capacity [12], and the finding was that microvascular dysfunction due to coronary endothelial dysfunction caused myocardial ischemia in patients with NOCAD owing to the overproduction of oxidative stress [12,75].

Seydi-Shirvani et al. reported an increase in malondialdehyde (MDA) levels and a significant decrease in levels of ferric reducing ability of plasma (FRAP), GSH levels, and SOD activity in patients with NOCAD, compared to a healthy control group [76]. MDA, a highly reactive three-carbon dialdehyde, is produced by the peroxidation of polyunsaturated fatty acid and is used as a marker of oxidative stress [77]. It is also produced by the metabolism of arachidonic acid, which is catalyzed by the enzyme, cyclo-oxygenase [78]. During oxidative stress, ROS triggers the peroxidation of membrane lipids, which results in the formation of MDA [77]. GSH levels and SOD activity are used to measure antioxidant markers and FRAP is used as a marker of total antioxidant capacity. FRAP refers to the capacity of antioxidant defense system to reduce or break down ROS. It can serve as an indicator of the susceptibility of antioxidants to the damage caused by oxidative stress. Changes in antioxidant levels reflect a compensatory mechanism used to counteract the increased levels of oxidative stress identified in patients with NOCAD [76]. Antioxidant defense mechanisms counteract the accumulation of ROS by scavenging and converting ROS to non-toxic molecules to reduce oxidative-stress-related damage. The contribution of oxidative stress to the development of NOCAD can also be evaluated by SOD activity. SODs have an important role in protection against lipid peroxidation and are recognized as the major antioxidant enzyme defense systems in the arterial vessel wall [76], and extracellular SODs are extracellular scavengers of superoxide anion in the vessel wall [62].

Ishii et al. suggested that Asian dust caused oxidative stress and inflammation associated with the development of myocardial infarction with non-obstructive coronary arteries (MINOCA) [63]. Asian dust, also known as yellow sand, is a meteorological phenomenon in East Asia, especially in spring. The dust originates in the deserts of East Asia and is spread by prevailing winds to China and Mongolia. Asian dust contains natural mineral soil, microorganisms, and anthropogenic hazardous components produced by factories that use fuels and by automobiles, and these components bind to the particles during transportation [79]. Short-term exposure to Asian dust is associated with a higher risk of MINOCA than obstructive CAD. 

The study by Ishii et al. in Japan assessed the association of admission of individuals with myocardial infarction with obstructive CAD and that of individuals with MINOCA to hospital after the occurrence two days earlier of Asian-dust events. The admission of individuals to hospital for MINOCA, after adjusting for meteorological variables, was found to correlate with Asian-dust events; however, a correlation was not established between the admission of individuals with myocardial infarction with obstructive CAD to hospital and the dust events. The absolute difference in the risk of MINOCA admission was 1.79 per 100,000 person-years [63]. The chemical analysis of Asian-dust-storm particles showed that the particles contained complex organic matter, elemental carbon, ammonium ions, sulfate ions, nitrate ions, and heavy metals, which may have contributed to oxidative stress [80].

In support of these findings, Chuang et al. proposed that the inhaled particles of urban air pollution induced systemic oxidative stress measured by 8-hydroxy-2′-deoxyguanosine, and the inhalation of polluted particles was also associated with cardiovascular events [81,82]. The findings of the study by Lee et al. further support the association between particulate matters (particle pollution) that cause oxidative stress and the induction of endothelial dysfunction and systemic inflammation, leading to cardiovascular events [83]. The study was conducted on community adults in a densely populated inner city neighborhood in Boston, Massachusetts, USA. The authors suggested that oxidative stress and systemic inflammation aggravated the adverse effects of fine particulate matter on cardiac autonomic function, even when the individuals were exposed to low pollutant levels [83].

Huang et al. reported that autonomic and vascular dysfunction increased cardiovascular-disease risk following exposure to high air-pollution levels [84]. Exposure to high levels of black carbon was established to be an oxidative-stress marker that independently correlated with major adverse cardiovascular events one month after exposure in patients presenting with acute coronary syndrome. Black carbon is a traffic-related particle that is a byproduct of combustion [85]. Therefore, populations that reside in urban and industrial areas are at a high risk of developing CAD, especially NOCAD.

An association was established in the study by Raad et al. between oxidative stress and diastolic dysfunction in patients with ischemia with no obstructive coronary artery disease (INOCA). An increase in aminothiol cysteine levels is often used as oxidative-stress marker, and it is associated with impaired endothelial function, increased arterial stiffness, carotid atherosclerosis, myocardial stiffness, and high rates of adverse cardiovascular events [45]. Cysteine thiols are significantly involved in oxidative-stress conditions. Cysteine is a primary aminothiol extracellular source that undergoes oxidation to form cysteine disulfide [86]. The study by Raad et al. showed that the role of oxidative stress in the pathogenesis of heart failure was not restricted to obstructive CAD and INOCA [45]. The authors suggested that systemic oxidative stress, measured by aminothiol cystine levels, was associated with diastolic dysfunction, even after adjusting for other underlying diseases that may have impacted diastolic function [45]. Oxidative stress has been shown to contribute to pathological hypertrophy, pathological remodeling, and the development and progression of cardiac failure [87,88]. In addition, the role of oxidative stress in heart failure was demonstrated in gene-transfer studies on primary antioxidant enzymes (SODs, catalase, and glutathione peroxidase), thioredoxin, and heme oxygenase-1 [87]. 

Ide et al. reported that left ventricular contractile failure was associated with oxidative stress. The increased production of ROS could have a role in the pathophysiology of heart failure. The study, conducted on adult mongrel dogs showed an increase in the production of hydroxyl radicals, originating from superoxide and hydrogen peroxide in the failing myocardium [89]. Thus, oxidative stress was seen to contribute to the development of NOCAD and to the pathogenesis of heart failure.

## 5. Burden of Non-Obstructive Coronary Artery Disease and Related Implications

Elsewhere, patients with NOCAD had higher rates of typical angina at follow-up, catheterization, and rehospitalization for unstable angina, as well as increased nitroglycerin use, compared to healthy individuals [99]. Groepenhoff et al. reported that clinic-based consultations between general practitioners and patients with non-obstructed coronary arteries were significantly higher for cardiovascular complaints (89%), compared with asymptomatic subjects (34%) [100]. In general, patients with NOCAD experience recurrent angina, which results in ongoing anxiety and a poor quality of life [22,101]. Individuals with NOCAD are also at high risk of cardiovascular adverse events and the associated socioeconomic impact and burden to individuals, families, and countries [102,103,104]. Factors that contribute to the pathogenesis of NOCAD, such as oxidative stress, should be addressed in treatment strategies that target this group of subjects, with a view to reducing the socioeconomic burden; in addition, they could constitute new potential predictors of prognosis and disease severity [105].

## 6. Conclusions

Oxidative stress can cause vasoconstriction and endothelial damage, resulting in coronary microvascular dysfunction and coronary vasospasm, which, in turn, leads to the pathogenesis of NOCAD. Although there is evidence from animal and human studies of the role of oxidative stress in cardiovascular disorders, antioxidant applications remain ineffective in preventing cardiovascular mortality. A better understanding of redox reactions and development of targeted antioxidants is important for the broad implementation of pharmacological therapies for cardiovascular diseases. Further advanced research on oxidative stress is needed to achieve this, especially to elucidate the role of oxidative stress in NOCAD.

## Figures and Tables

**Table 1:** Reports on oxidative stress in CAD.

Parameters	Findings	References
Thioredoxin	Glucose intolerance with CAD ^1^ was linked to high levels of thioredoxin, an oxidative stress marker.	[66]
MDA ^2^	The plasma levels of MDA ^2^-modified LDL ^3^ were significantly higher in patients with acute coronary syndrome than patients with stable CAD ^1^, and this correlated with increased levels of troponin I and C-reactive protein.	[90]
Aminothiols, cystine, glutathione, and cystine/glutathione ratio	High burdens of oxidative stress were quantified by plasma aminothiols, cystine, glutathione, and their ratio, was associated with mortality in patients with CAD ^1^	[91]
CyPA ^4^	Plasma CyPA ^4^ level was a novel biomarker for oxidative stress and CAD ^1^ in humans.	[92]
MDA ^2^, O_2_, and SOD ^5^ activity	Level of MDA ^2^ and O_2_− in plasma were significantly higher with lower level of SOD ^4^ activity in patients with CAD ^1^.	[93]
Ox-LDL ^6^	Ox-LDL ^6^, ox-LDL ^6^/total cholesterol, ox-LDL ^6^/HDL-C ^7^, ox-LDL ^6^/LDL-C ^8^, and ox-LDL ^6^/albumin levels were significantly high in CAD ^1^ patients and subjects with hypertension and/or diabetes.	[94]
Lipid hydroperoxide, total antioxidant status, total oxidant status, oxidative stress index, paraoxonase, and arylesterase activities	High oxidative stress index values and elevated total oxidant status levels were associated with disease severity, vascular damage and there was strong correlation with heavy smoking in the early development of CAD ^1^	[95]
MDA ^2^ and erythrocytes SOD ^4^ activity	An increase in MDA ^2^ plasma levels and a corresponding decrease in glutathione and glutathione peroxidase levels may serve as potential biomarkers for detecting the early development of atherosclerosis.	[96]
Ox-LDL ^5^	High ox-LDL ^6^ levels showed a significant positive correlation with the severity of acute coronary syndrome. Severe lesions contained a significantly higher percentage of ox-LDL ^6^-positive macrophages, suggesting that increased levels of ox-LDL ^6^ correlated with plaque instability in coronary atherosclerotic lesions.	[97]
Platelet aggregation, MDA ^2^, plasma-ionized Ca21, and antioxidant enzymes (glutathione peroxidase and SOD ^4^)	Platelet aggregation and the plasma levels of MDA ^2^ and plasma-ionized Ca21 increased significantly in patients with CAD ^1^, compared to the control group. A decrease in antioxidant enzymes activity was observed with the exception of a slight increase in glutathione peroxidase levels in patients with myocardial infarction.	[98]

^1^ CAD, coronary artery disease; ^2^ MDA, malondialdehyde; ^3^ LDL, low-density lipoprotein; ^4^ SOD, superoxide dismutase; ^5^ CyPA, plasma of cyclophilin A; ^6^ ox-LDL, oxidized low-density lipoprotein; ^7^ HDL-C, high-density lipoprotein–cholesterol, ^8^ LDL-C; low-density lipoprotein–cholesterol.

**Table 2:** Reports on oxidative stress in NOCAD.

Parameters	Findings	References
Total oxidant status, lipid hydroperoxide levels and oxidative stress index	Oxidant levels were high in patients with cardiac syndrome X.	[12]
MDA ^1^, GSH ^2^, and SOD ^3^ activity	An increase in the levels of MDA ^1^, an oxidative stress marker, along with a decrease in the levels of major important antioxidants, such as FRAP ^4^ and GSH ^2^, and a reduction in SOD ^3^ activity was demonstrated in patients with cardiac syndrome X, compared to the healthy controls.	[76]
Asian dust	Short-term exposure to Asian dust was associated with a high risk of MINOCA ^5^.	[63]
Cystine	Cystine levels increased in patients with INOCA ^6^.	[45]

^1^ MDA, malondialdehyde; ^2^ GSH, glutathione; ^3^ SOD, superoxide dismutase; ^4^ FRAP, ferric reducing ability of plasma; ^5^ MINOCA, myocardial infarction with no obstructive coronary artery disease; ^6^ INOCA, ischemia with no obstructive coronary artery disease.

## Abbreviations

CAD	Coronary artery disease
CyPA	Plasma cyclophilin A
FRAP	Ferric reducing ability of plasma
GSH	Glutathione
HDL-C	High-density lipoprotein–cholesterol
HNE	4-hydroxynonenal
INOCA	Ischemia with no obstructive coronary artery disease
LDL	Low-density lipoprotein
LOOH	Lipid hydroperoxide level
MDA	Malondialdehyde
MINOCA	Myocardial ischemia with no obstructive coronary artery disease
NO	Nitric oxide
NOCAD	Non-obstructive coronary artery disease
OX-LDL	Oxidized low-density lipoprotein
ROS	Reactive oxygen species
SOD	Superoxide dismutase
